# Neural Sequence Generation Using Spatiotemporal Patterns of Inhibition

**DOI:** 10.1371/journal.pcbi.1004581

**Published:** 2015-11-04

**Authors:** Jonathan Cannon, Nancy Kopell, Timothy Gardner, Jeffrey Markowitz

**Affiliations:** 1 Department of Mathematics, Boston University, Boston, Massachusetts, United States of America; 2 Department of Biology, Brandeis University, Waltham, Massachusetts, United States of America; 3 Department of Biology, Boston University, Boston, Massachusetts, United States of America; Indiana University, UNITED STATES

## Abstract

Stereotyped sequences of neural activity are thought to underlie reproducible behaviors and cognitive processes ranging from memory recall to arm movement. One of the most prominent theoretical models of neural sequence generation is the synfire chain, in which pulses of synchronized spiking activity propagate robustly along a chain of cells connected by highly redundant feedforward excitation. But recent experimental observations in the avian song production pathway during song generation have shown excitatory activity interacting strongly with the firing patterns of inhibitory neurons, suggesting a process of sequence generation more complex than feedforward excitation. Here we propose a model of sequence generation inspired by these observations in which a pulse travels along a spatially recurrent excitatory chain, passing repeatedly through zones of local feedback inhibition. In this model, synchrony and robust timing are maintained not through redundant excitatory connections, but rather through the interaction between the pulse and the spatiotemporal pattern of inhibition that it creates as it circulates the network. These results suggest that spatially and temporally structured inhibition may play a key role in sequence generation.

## Author Summary

Sequences of stereotyped actions are central to the everyday lives of humans and animals. It was hypothesized over half a century ago that these behaviors were enabled by linking together groups of neurons (or “cell assemblies”) into a feedforward chain using correlation-based learning rules. These chains could then be activated to generate particular behavioral sequences. However, recent data from HVC (the songbird analogue of premotor cortex) paint a more complicated picture: inhibitory and excitatory cells lock to different phases of a rhythm, with inhibitory cells providing windows of opportunity for the excitatory cells to fire. This study puts forward a mathematical model that uses both a feedforward chain geometry and local feedback inhibition to generate stereotyped neural sequences. The chain conducts an excitatory pulse through multiple spatial regions, arriving at each as local inhibition dips. Our simulations and analysis demonstrate that such patterned local inhibition can synchronize the firing of pools of neurons and stabilize spike timing along the chain. Our model provides a new way of thinking about sequence generation in the songbird and in neural circuits more generally.

## Introduction

From the kingfisher’s dive to the performance of a piano concerto, sequences of stereotyped actions are central to the everyday lives of humans and animals. One of the most well-studied behavioral sequences in nature is birdsong, and the physiology underlying HVC (the songbird analogue of mammalian premotor cortex and presumed neural sequence generator) has been a topic of intense interest. Principal neurons in HVC produce sparse, time-locked bursts of activity that are stereotyped from trial to trial [1, 2]. Temporally-ordered neural activity has also been observed in other species in the context of various sequential behaviors [3–5], but the extreme precision and sparsity of the songbird premotor projection cells in HVC are unmatched. How is this spike timing precision maintained in the presence of biological noise? What makes neural sequence generation in this context robust?

**Fig 1:**
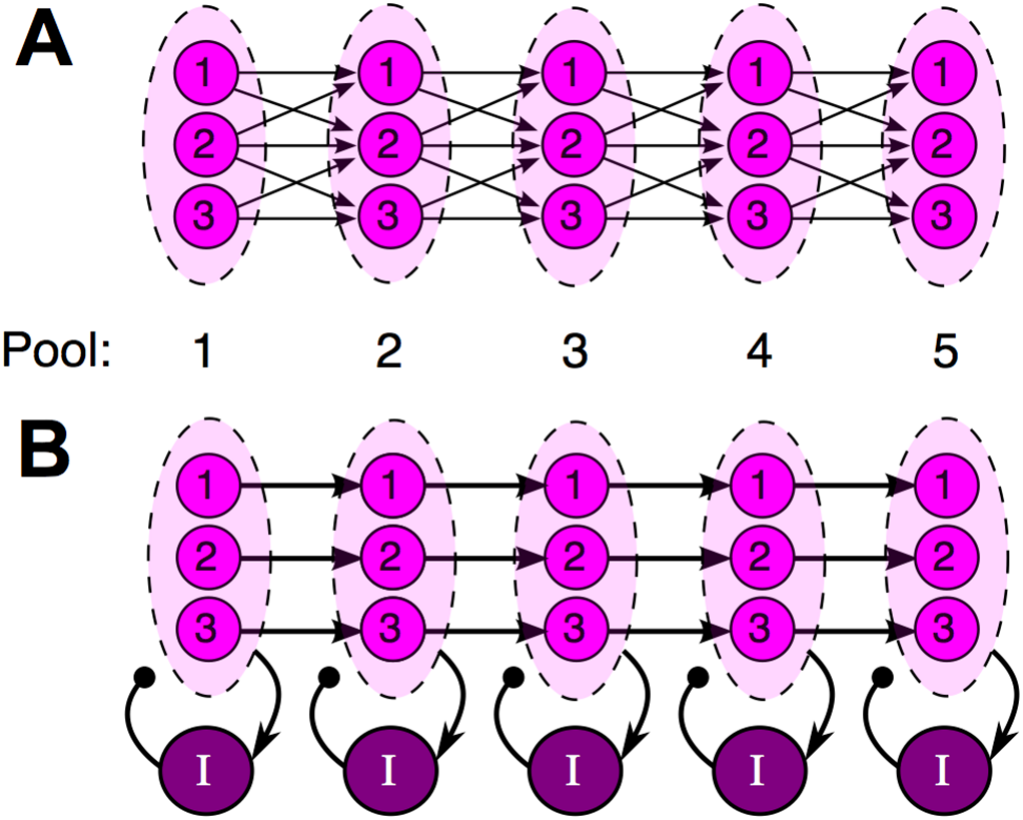
A schematic comparing the synfire chain (A) to our proposed model (B). **A**, The standard synfire chain. Fan-in and fan-out connectivity allows the spike timing of multiple cells in one pool to influence the spike timing of each cell in the next, which ultimately leads to synchronized spiking within pools. **B**, Parallel chains generate sequences simultaneously with shared feedback inhibition. Cells in each pool share no common excitatory input and do not directly interact with each other. Instead, synchrony is promoted by inhibitory feedback shared by all cells in a pool. When the interneurons are activated repeatedly by spatially recurrent excitatory activity, this common inhibitory input proves sufficient to synchronize spiking within pools (see Fig 2).

A well-studied model that provides a possible answer is the synfire chain [6, 7]. The synfire chain assumes that excitatory (principal) neurons are organized into pools arranged in a redundant feedforward geometry (Fig 1A). Although synfire chains have not been directly observed in HVC, simulations have shown that the redundant synfire geometry combined with a threshold non-linearity can generate stereotyped, precisely timed sequential activity similar to that observed experimentally in HVC.

Typically, models based on the synfire chain either exclude inhibition entirely [8], or simply use global inhibition to prevent runaway excitation or select between competing chains [9, 10]. But a series of recent findings in zebra finch suggest that inhibitory spiking plays a larger role in defining the timing of excitatory cell sequences HVC than has been previously assumed. Guitchounts et al. [11] find that inhibitory spiking in HVC, like excitatory spiking, is extremely stereotyped from trial to trial. Kosche et al. [12] find that principal cells that project to the vocal motor pathway (HVC_RA_) receive stereotyped excitatory inputs at multiple points during the song, but only burst when excitatory inputs align with pauses in inhibition. And Markowitz et al. [13] recently observed that in a given region of HVC, excitatory cells that project to the basal ganglia (HVC_X_) and inhibitory cells (HVC_I_) fired at distinct phases of a stereotyped 30Hz component of the local field potential. Local field potentials were not globally synchronized across HVC; instead, their phase varied over the spatial extent of HVC, suggesting that the phasic coordination of cell firing was local rather than global.

Here, we describe a general spiking model of neural sequence generation inspired by the observations of Markowitz et al. [13] that shows a similar local alternation of excitatory and inhibitory activity. Like the standard synfire chain, the model is based on feedforward excitatory chains that define an ordered sequence of cell firing. However, in contrast to the synfire chain, the separate strands of the chain do not coordinate their simultaneous activity through crossing excitatory connections. Rather, the strands interact and synchronize through common feedback inhibition (Fig 1B), which produces local inhibitory cycles that act as spatiotemporal “scaffolding” for the feedforward excitatory activity of the chain. We demonstrate in simulation that this locally patterned feedback inhibition synchronizes pools of cells like a synfire chain, and we present analysis that quantitatively describes this effect in terms of the decay rate of inhibition and other system parameters. Our simulations also show that our proposed network and its local inhibitory dynamics help to control the drift of spike timing from one trial to the next.

Unlike many previous HVC modeling efforts, [14–16], our model is not intended to describe HVC in biological detail; instead, we use it to illustrate and investigate the possible contribution of local feedback inhibition to sequence generation. We do, however, discuss intriguing correspondences between the model’s behavior and observations in HVC.

## Models

### General model

**Fig 2:**
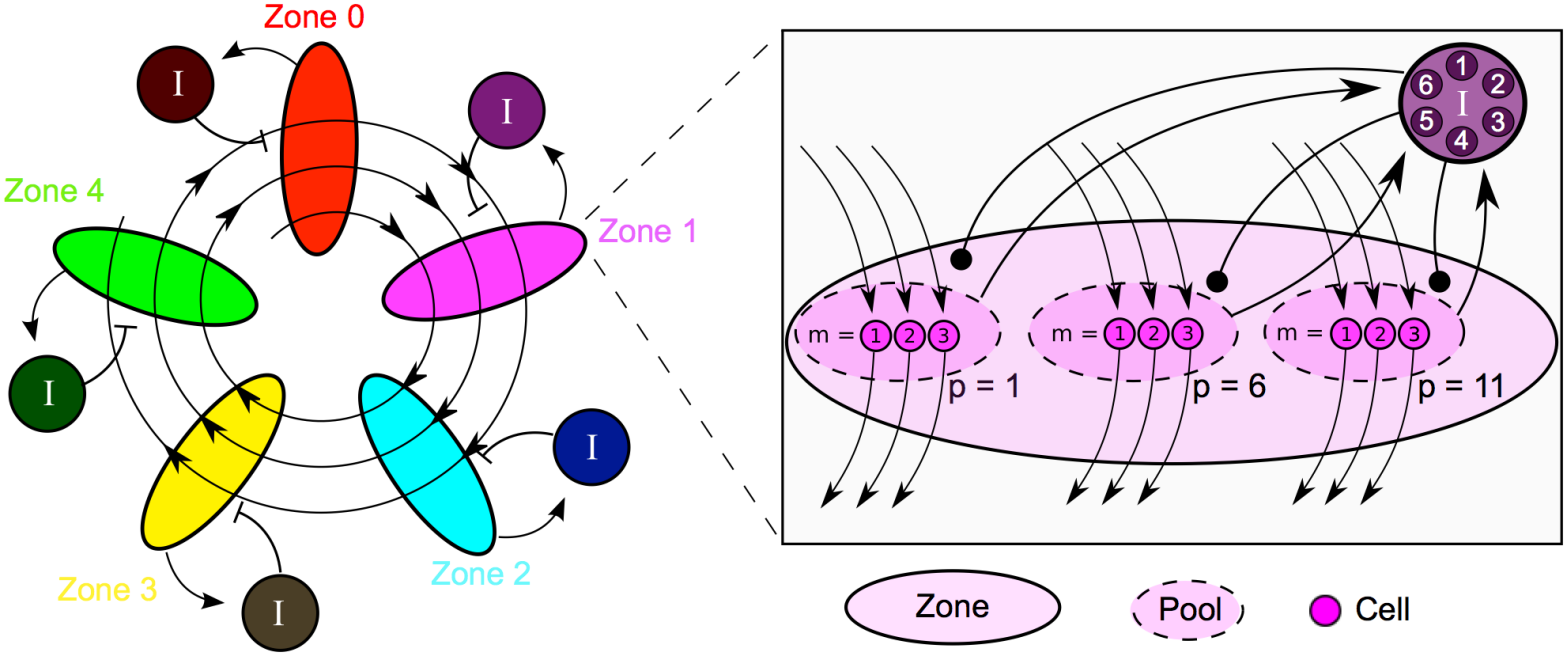
A detailed schematic of the proposed model. The model is pictured with *N* = 5 inhibitory zones, *P* = 3 pools of excitatory cells per zone, *M*_*e*_ = 3 principal cells in each pool, and *M*_*i*_ = 6 inhibitory cells in each zone. **Left**, A chain of activity circulates repeatedly through the inhibitory zones. Excitatory activity in a zone excites a collection of inhibitory cells shared by all principal cells in the zone. **Right**, The fine structure of the cells in zone 1. The chain of activity consists of the firing of a sequence of near-synchronous “pools” of *M* = 3 cells. Pools are numbered in order of firing. Since the chain of pools circulates through the zones, pools 1, 6, and 11 are in zone 1, and, more generally, pool *p* is in zone *p* mod *N*. Cell *m* in pool *p* receives excitatory input from cell *m* in pool *p* − 1 and feeds forward to cell *m* in pool *p* + 1, forming *M* parallel sub-chains. The spiking of the cells in a pool excites the inhibitory cells shared by all pools in their zone.

In this work, a model of sequence generation is presented in which a spiraling excitatory chain conducts a pulse of excitatory activity repeatedly through multiple “zones” of inhibition (Fig 2). In each zone, the arrival of the pulse causes a pool of principal cells to fire; these cells then excite both a pool of principal cells in the next zone and a local source of feedback inhibition. The inhibition then decays until the pulse returns to that inhibitory zone. We show that the presence of this decaying inhibition acts to synchronize the firing of other local excitatory cells when the pulse returns and helps to establish spike timing consistency from one trial to the next.

In order to demonstrate that synchronization in our model is independent of the synchronizing effect of synfire connectivity, we intentionally structure our excitatory chain without any fan-in or fan-out connectivity between consecutive pools of excitatory cells. Instead, each cell in a pool sends an excitatory projection to exactly one cell in the next pool. This connectivity pattern creates multiple parallel excitatory strands that do not interact through excitation—the only interaction between these strands is moderated by the local feedback inhibition activated when the pulse passes through each inhibitory zone (Figs 1B and 2). The conceptual model presented here can also be implemented with excitatory pools connected in a synfire chain, but such an implementation would not serve our purpose of demonstrating that synchronization in our model is independent of the synfire mechanism.

### Specific implementation

The model described here was implemented in MATLAB. All code is available in a figshare digital repository at http://dx.doi.org/10.6084/m9.figshare.1570957.

All excitatory and inhibitory cells are modeled by quadratic integrate-and-fire (QIF) neurons with white noise [17]. The voltage *V* of a QIF neuron evolves according to the stochastic differential equation

$$CdV=(\frac{V^{2}}{R}+I\left(t\right)\;)\;dt+DdW\left(t\right)$$ (1)

where *C* is the neuron’s membrane capacitance, *R* is the resistance associated with the leak current, *W*(*t*) is a white noise process with variance 1, *D* is the amplitude of voltage noise, and *I*(*t*) is a source of time-varying external drive to the neuron. (All quantities are without units.) In our model, *I*(*t*) is a sum of excitatory and inhibitory post-synaptic currents (EPSCs and IPSCs, respectively) and a constant level of tonic drive. When *V* reaches a specific spiking voltage *V*_*S*_, it resets to a reset voltage *V*_*R*_ < *V*_*S*_. In our model, *V*_*S*_ = 1. For the inhibitory cells, *V*_*R*_ = −1. We are interested in the synchronization of a single pulse that activates each excitatory cell only once (emulating the sparse firing of RA-projecting cells in HVC), so once the voltage of an excitatory cell reaches *V*_*S*_, it is no longer recorded.

All cells are divided between *N* inhibitory zones. Each zone contains *P* pools of *M*_*e*_ principal cells. Pools are ordered and numbered 0, …, *NP* − 1 in a chain that spirals through the zones *P* times so that pool *p* belongs to zone (*p* mod *N*). The principal cells in pool *p* are numbered *m* = 0, …, *M*_*e*_ − 1. Cell *m* in pool *p* projects one-to-one to cell *m* in pool *p* + 1, forming a spiraling chain composed of *M*_*e*_ parallel strands.

When the system is initialized, spike times are chosen for cells in pool zero. For *p* > 0, cell *m* in pool *p* is modeled by a QIF neuron with voltage $V_{p}^{m}$. We let $t_{p}^{m}$ denote the firing time of excitatory cell *m* in pool *p*. At this time, an EPSC is initialized in cell *m* in pool *p* + 1. The temporal profile of an EPSC in an excitatory cell is *g*_*ee*_ *E*(*t*), where *g*_*ee*_ is the strength of excitatory-to-excitatory connections and *E*(*t*) is the evolution of a gating variable over time. We require that *E*(*t*) be a positive, continuous function with *E*(*t*) = 0 for *t* ≤ 0 and *E*(*t*) differentiable for *t* > 0. Since the EPSC in cell *m* in pool *p* is initialized at time $t_{p}^{m}$, its height at time *t* is $g_{ee}E\left(t-t_{p}^{m}\right)$. An additional tonic drive of magnitude *I*_*E*_ is applied to each principal cell.

Every zone *n* contains a collection of *M*_*i*_ inhibitory QIF neurons with voltages $U_{n}^{m}$ for *m* = 0, …, *M*_*i*_ − 1. These neurons are excited by the firing of any excitatory cell in any pool in zone *n*: when an excitatory neuron fires and initializes an EPSC in its downstream excitatory cell, it also delivers an EPSC to all inhibitory cells in its zone. This EPSC is described by the function $\frac{g_{ei}}{M_{e}}E\left(t-t_{p}^{m}\right)$, where *g*_*ei*_ is the strength of excitatory-to-inhibitory connections. The excitatory drive to each inhibitory cell is $\frac{g_{ei}}{M_{e}}\sum_{p\;\text{in}\;\text{zone}\;n}\sum_{m=0}^{M_{e}-1}E\left(t-t_{p}^{m}\right)$, the sum of the EPSCs it has received from all excitatory cells in its zone.

When an inhibitory cell fires in zone *n*, a sustained IPSC is delivered to all excitatory and inhibitory cells in that zone. A single inhibitory synaptic gating variable *ϕ*_*n*_ is incremented by $\frac{k}{M_{i}}$ (for some *k* > 0) each time a local inhibitory cell spikes, and decays exponentially with time constant *T*_*i*_ between spikes. Inhibition affecting excitatory and inhibitory cells in zone *n* is *ϕ*_*n*_ scaled by conductances *g*_*ie*_ and *g*_*ii*_, respectively.

Substituting a sum of excitation and inhibition for *I*(*t*) in Eq (1), we have the following equations for excitatory and inhibitory neuron membrane potentials ($V_{p}^{m}$ and $U_{n}^{m}$, respectively):

$$\begin{array}{cc} C_{e}dV_{p}^{m} & =\left(\frac{{\left(V_{p}^{m}\right)}^{2}}{R_{e}}+g_{ee}E\left(t-t_{p-1}^{m}\right)-g_{ie}\phi_{\left(p\text{ mod }N\right)}+I_{E}\right)\;dt+D_{e}dX_{p}^{m}\left(t\right)\\ C_{i}dU_{n}^{m} & =\left(\frac{{\left(U_{n}^{m}\right)}^{2}}{R_{i}}+\frac{g_{ei}}{M_{e}}\underset{p\text{ in zone }n}{\sum }\overset{M_{e}-1}{\underset{m=0}{\sum }}E\left(t-t_{p}^{m}\right)-g_{ii}\phi_{n}\right)\;dt+D_{i}dW_{n}^{m}\left(t\right)\\ d\phi_{n} & =\left(-\frac{\phi_{n}}{T_{i}}+\frac{k}{M_{i}}\underset{s}{\sum }\delta \left(t-t_{n}^{s}\right)\right)\;dt \end{array}$$ (2)

where $dX_{p}^{m}\left(t\right)$ and $dW_{n}^{m}\left(t\right)$ are white noise processes with variance 1; $\left\{t_{n}^{s}\right\}$ is the set of local inhibitory spike times, i.e., times that $U_{n}^{m}=1$ for any *m*, indexed by *s*; and $\delta (t-t_{n}^{s})$ is a Dirac delta function that integrates to 1 at any local inhibitory spike time.

### Simulation initialization and parameters

This system must be initialized in simulation from a set of initial voltages $V_{p}^{m}$ and $U_{n}^{m}$, a set of *M*_*e*_ initial excitatory spike times $t_{0}^{m}$, and a set of *N* gating variables *ϕ*_*n*_ that determine the initial level of inhibition in each zone at time *t* = 0. All voltages were initialized from zero at *t* = 0. Initial excitatory spike times were set by drawing $t_{0}^{m}$ from a Gaussian distribution with mean 0ms and variance 2ms^2^. Inhibitory gating variables *ϕ*_*n*_ were initialized at a constant *ϕ* ^0^.

Parameters were chosen for this model in order to produce the desired dynamics when local feedback was activated. Specifically, the model was built and tuned with the following objectives in mind:

- Most or all of the pool had to fire before most or all of the local inhibitory response. Thus, the firing of a pool of principal cells had to evoke local feedback inhibition with a sufficiently large delay. In order to implement this delay, we chose inhibitory cell membrane capacitance and resistance relatively large, slowing the response (and, in particular, the membrane potential rise time) of the inhibitory cells. A swifter inhibitory response, as might have been produced by cell with shorter membrane time constants or, e.g., leaky integrate-and-fire neurons, would have produced competition between principal cells within the pool (see, e.g., [18] or [19]), which could play an important role in a sequence generating circuit of this type but was outside the scope of our study.
- At each spike volley, the decay of the inhibition produced by the previous local volley had to still be in progress. Thus, we had to choose an inhibitory decay time constant *T*_*i*_ that agreed roughly with the amount of time it took for an excitatory pulse to circle the loop once. If *T*_*i*_ was too large, inhibitory decay was too slow to produce noticeable synchronizing effects; if it was too small, the inhibition would be almost entirely gone by the time the pulse returned, with similar results.
- Synaptic conductances had to be tuned such that excitatory volleys consistently evoked responses in downstream excitatory and inhibitory cells, even when those cells were partially inhibited. As we note in the Discussion, propagation failure due to inhibition could help control for relaxed architectural constraints; however, pulse propagation failure was also outside the scope of our study.
- For feedback inhibition to improve the consistency of spike volley timing across trials, the response of the inhibitory populations to local spike volleys had to be consistent across trials. Running the simulation with a large number of inhibitory cells helped average out the effects of noise on the inhibitory population: when *M*_*i*_ was set to 1, we did not observe improved timing across trials.

As long as these four conditions were met, the effects of local feedback inhibition on spike volley synchronization and timing described below were robust to variation in model parameters.

We simulated this system with two different sets of parameters. In simulation 1, we set *M*_*e*_ = 20, *M*_*i*_ = 50 and *N* = 5, and simulated the system with and without feedback inhibition. As we discuss below, this simulation run with feedback inhibition produced short periodic volleys of inhibitory spikes in each zone, an activity pattern considerably tidier than that of inhibitory cells observed in HVC. These dynamics obeyed conditions that made the system analytically tractable, allowing us to provide a quantitatively accurate theory explaining our simulation results. However, we also wanted to show that a more complex pattern of inhibitory activity could produce qualitatively similar results. For this purpose, we performed simulation 2, for which we set *M*_*e*_ = 50 and adjusted various parameters related to the inhibitory cells. All parameter values for both simulations are listed in Table 1.

**Table 1:** Parameter values.

	Simulation 1	Simulation 2
*M*_*e*_	20	50
*M*_*i*_	50	50
*N*	5	5
*P*	20	10
*C*_*e*_	0.3	0.3
*C*_*i*_	1	1
*R*_*e*_	2	2
*R*_*i*_	4	2
*D*_*e*_	0.2	0.2
*D*_*i*_	0.1	0.2
*T*_*i*_	30ms	20ms
*k*	0.5	0.5
*ϕ* ^0^	1	0.5
*g*_*ei*_	0.6	0.4
*g*_*ee*_	1	1
*g*_*ii*_	0.2	0
*g*_*ie*_	0 / 0.3	0 / 0.3
*I*_*E*_	-0.3 / -0.15	-0.3 / -0.15

Parameters used in simulations 1 and 2. Values separated by slashes are given for simulations without / with feedback inhibition.

**Fig 3:**
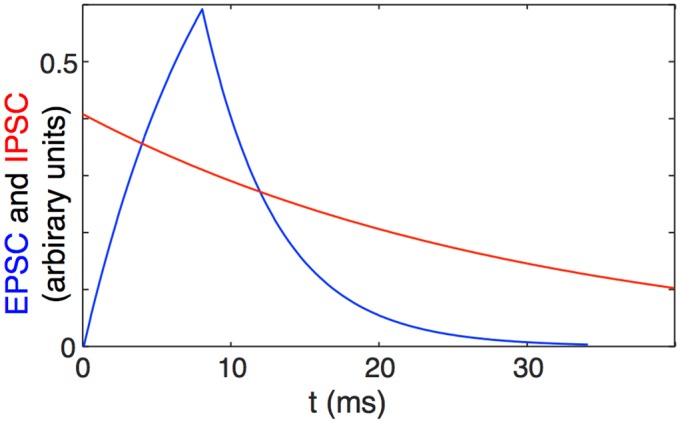
Example EPSC and IPSC. The function E(t) defined in Eq (3) and used in simulation to model an EPSCs (blue), compared with the decay rate of an IPSP in simulation (red).

For our simulations, we chose a simple but biologically-motivated function *E*(*t*):

$$E\left(t\right)=\{\begin{array}{cc} 0 & \;\text{for}\;t<0\\ \left(1-e^{-\frac{t}{\tau_{r}}}\right) & \;\text{for}\;0\leq t<r\\ \left(1-e^{-\frac{r}{\tau_{r}}}\right)e^{-\frac{t-r}{\tau_{d}}} & \;\text{for}\;r\leq t \end{array}$$ (3)

where *τ*_*r*_ is the time constant for the rise of the EPSC, *r* is the duration of its rise, and *τ*_*d*_ is the time constant of its decay (Fig 3). We set *τ*_*r*_ = 9ms, *τ*_*d*_ = 5ms, and *r* = 8ms. We chose long rise times to mimic the ≈ 10ms duration of principal cell bursts in HVC [1] and the ≈ 10ms depolarizations observed in these cells and attributed to principal-to-principal cell excitatory potentials [2].

When we ran simulation 1 with no feedback inhibition, we set *g*_*ie*_ = 0 and *I*_*E*_ = −0.3 in Eq (2) such that the level of drive to the excitatory cells was only the sum of a constant background and any incoming EPSC. We ran this simulation from the initial conditions described above. When we ran simulation 1 with feedback inhibition and simulation 2, we set *g*_*ie*_ = 0.3. This change increased the average level of inhibition, so we offset its effect by raising *I*_*E*_ to −0.15.

## Results

### Spatiotemporally patterned dynamics

**Fig 4:**
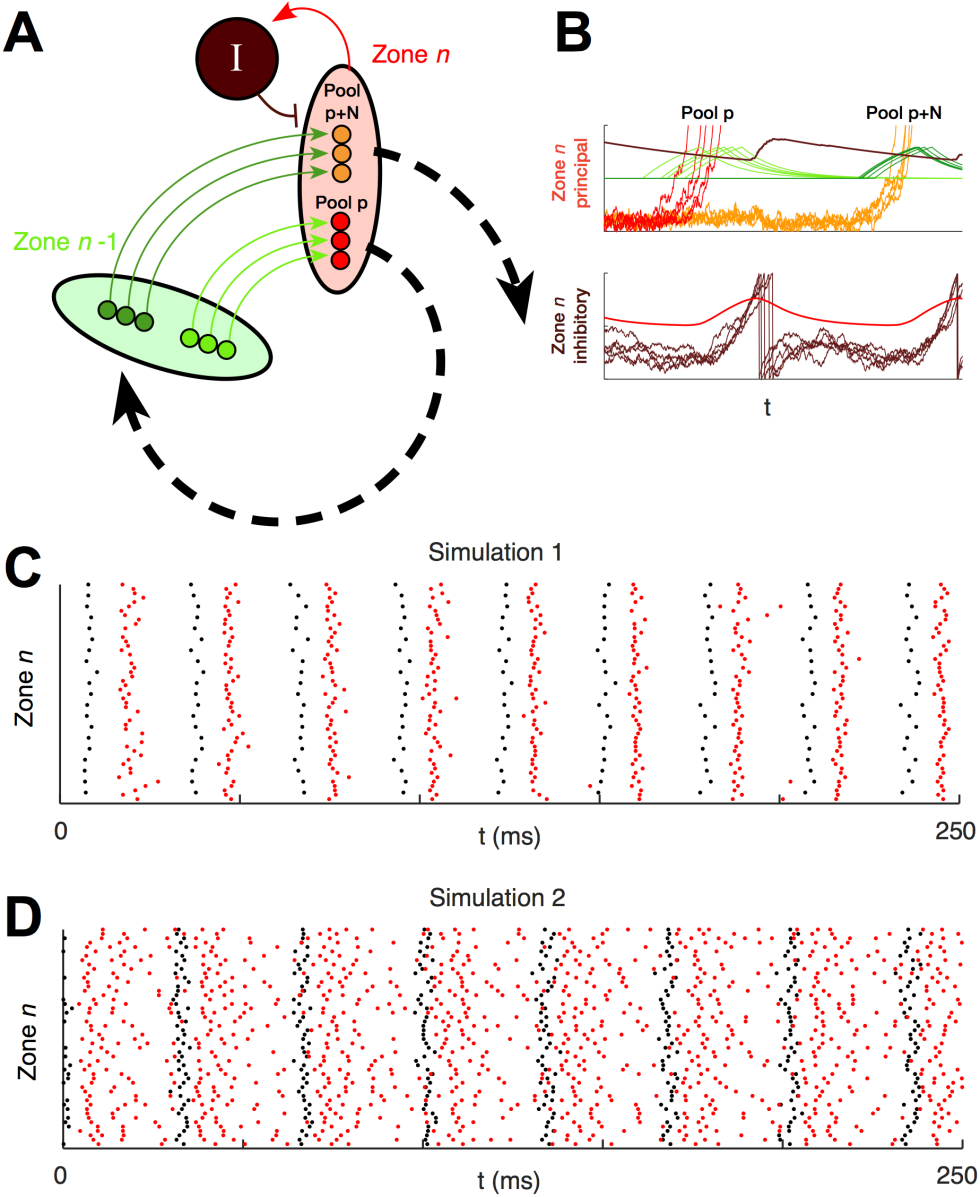
Local temporal coordination between an excitatory pulse and feedback inhibition. **A**, Schematic illustration of two zones of the network. **B**, Voltage and current traces from simulation 1 with feedback inhibition. Above, voltages of principal cells *m* = 1, …, 5 in pools *p* and *p* + *N* in zone *n*, their respective afferent EPSCs $E(t-t_{p-1}^{m})$ and $E(t-t_{p+N-1}^{m})$, and local inhibition *ϕ*_*n*_ are displayed on the same unit-less y-axes ranging from −1 to 1. Below, voltages of inhibitory cells *m* = 1, …, 5, in zone 0 and their afferent excitation $\sum_{p\;\text{in}\;\text{zone}\;n}\sum_{m=0}^{M_{e}-1}E\left(t-t_{p}^{m}\right)$ are similarly displayed. Colors correspond to the schematic in **A**. On the upper axes, bright red is ${\text{V}}_{\text{P}}^{\text{m}}$ for all cells *m* in pool *p*; orange is the same for pool *p+N*; light green is the EPSPs created in pool *p* by cells in pool *p-1*; dark green is the EPSPs created in pool *p+N* by cells in pool *p+N-1*; and dark red is g_ie_ϕ_n_, the level of local I-to-E inhibition. On the lower axes, dark red is ${\text{U}}_{\text{n}}^{\text{m}}$ for all inhibitory cells *m* in zone *n*, and bright red is the net E-to-I excitation affecting these cells. Principal cell spiking in pool *p* excites local inhibitory cells, which respond by spiking and elevating levels of inhibition to the local principal cells. The next local pool, pool *p* + *N*, spikes when the excitatory pulse has circled the network and returned. The pulse arrives as the local inhibition decays. **C**, A spike raster from one zone in simulation 1 with feedback inhibition shows local alternation of volleys of principal cell spikes (black) and volleys of inhibitory spikes (red). **D**, A spike raster from one zone in simulation 2 with feedback inhibition shows local inhibitory spiking (red) continuing throughout the simulation, with periodic volleys of local principal cell spikes (black) alternating with periods of increased inhibitory firing rate.

**Fig 5:**
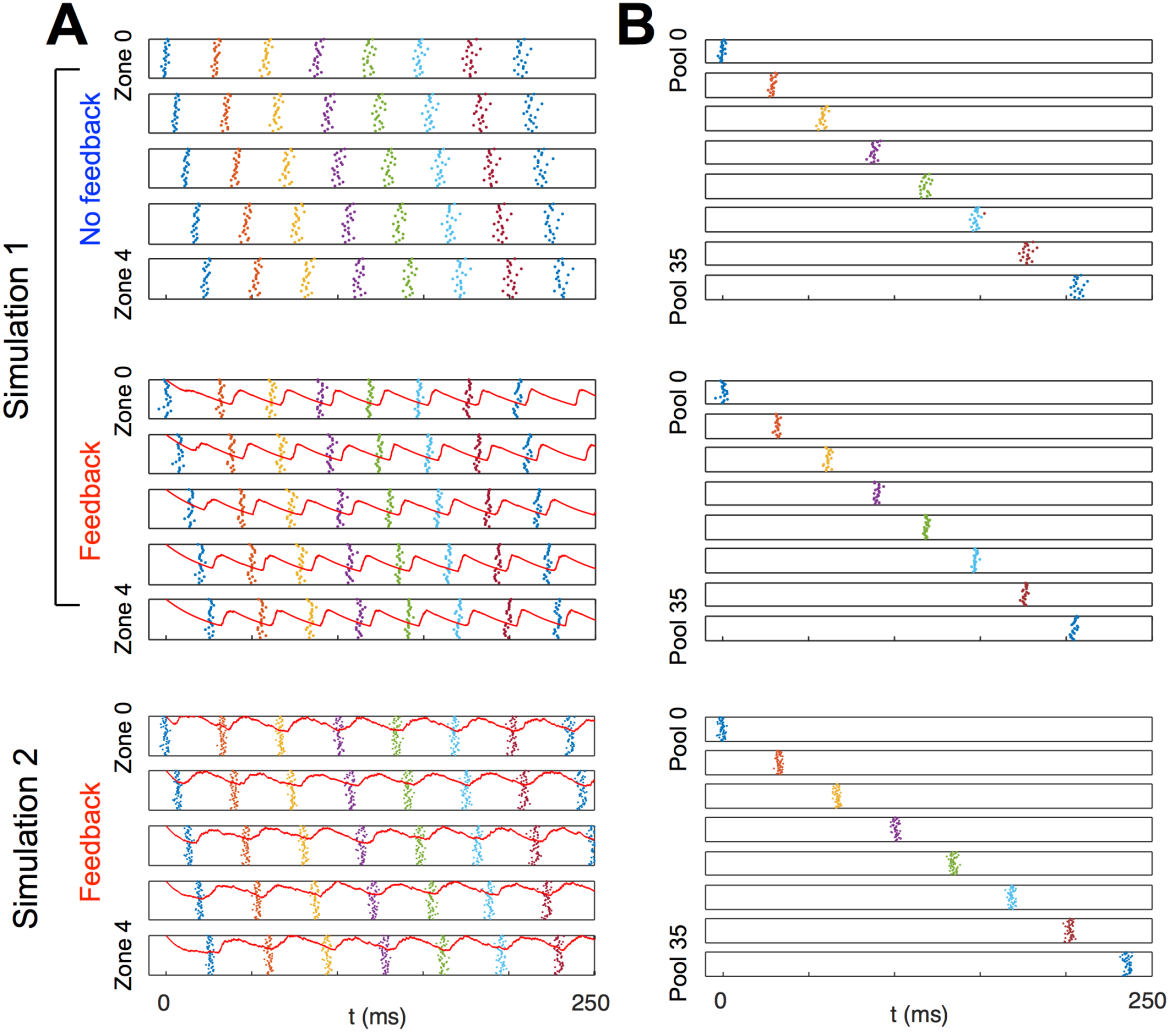
Simulation demonstrates the synchronizing effect of local feedback inhibition. Feedback inhibition is provided by 50 feedback inhibitory interneurons in each inhibitory zone. Results are displayed for simulation 1 without feedback, and for both simulations with feedback. **A**, Rasters of the first 40 excitatory spike volleys, sorted by inhibitory zone. Zones are vertically stacked by zone index *n*, and cells in each zone are vertically stacked by index *m*. Note that each spike volley is created by a different pool of cells. Thus, the first spike volley on the first row represents the spiking of pool 0, the second represents the spiking of pool 5, etc., and the topmost row of spikes are the spikes of cell *m* = 0 in each of these pools. In both simulations with feedback, local inhibition (red trace) rises due to the firing of local inhibitory cells shortly after each excitatory spike volley. This local inhibition is still decaying when the next spike volley occurs in the same zone. **B**, Rasters of spike volleys of the first eight pools in inhibitory zone 0, with and without feedback inhibition. Each row represents a pool of *M*_*e*_ = 20 excitatory cells, each on one of *M*_*e*_ parallel subchains. Pools are vertically stacked in the order in which they receive the excitatory pulse, i.e., in order of their pool index *p*; cells in each pool are vertically stacked by index *m*. In simulation 1 without feedback inhibition, the parallel strands of the chain do not interact, so pools of cells do not synchronize. In both simulations with feedback inhibition, the pool attain and maintain a level of approximate synchrony, though the effect is more dramatic in simulation 1.

Our model produced a spatiotemporal pattern of coordinated excitation and inhibition. In each region, the periodic arrival of the excitatory pulse triggered a periodic local inhibitory feedback response; thus, a wave of blanket inhibition circulated among the regions following the excitatory pulse. This pattern created a local alternation between excitatory and inhibitory firing (Fig 4), reminiscent of the observation of cell-type specific phase preferences in the 30Hz component of the local field potential in HVC [13]. In simulation 1, sharp volleys of principal cell spikes alternated with sharp volleys of inhibitory cell spikes (Fig 4B and 4C). In simulation 2, more inhibitory cells were included in each zone, inhibition between inhibitory cells was eliminated, and additional noise was added to inhibitory cell membrane potentials. In this simulation, inhibitory cells fired at all phases of the local cycle, but their firing rates increased after each local excitatory pool spiked and decreased again before the pulse returned to the same zone (Fig 4D). Instead of excitatory and inhibitory spikes occurring in short, discrete, alternating volleys, excitatory spikes occurred during phases of reduced inhibitory spiking, resembling the excitatory spiking during pauses in inhibition observed by Kosche et al. [12]. Our simulations also agreed with the observation of Markowitz et al. [13] that the phasic coordination of principal cells and inhibitory cells was not global: locally, excitatory spiking was locked to an inhibitory cycle, but globally, excitatory spiking continued throughout that cycle (Fig 5A).

### Synchronization

Upon examining individual excitatory spike rasters from our simulations (Fig 5), it was clear that local feedback inhibition promoted synchrony within pools of principal cells. Without feedback, there was no interaction between the *m* parallel strands of the chain, so the independent sources of noise caused the spike times within pools to drift apart; with feedback, the distribution of spike times instead remained tight as spiking propagated along the chain and around the ring of inhibitory zones.

**Fig 6:**
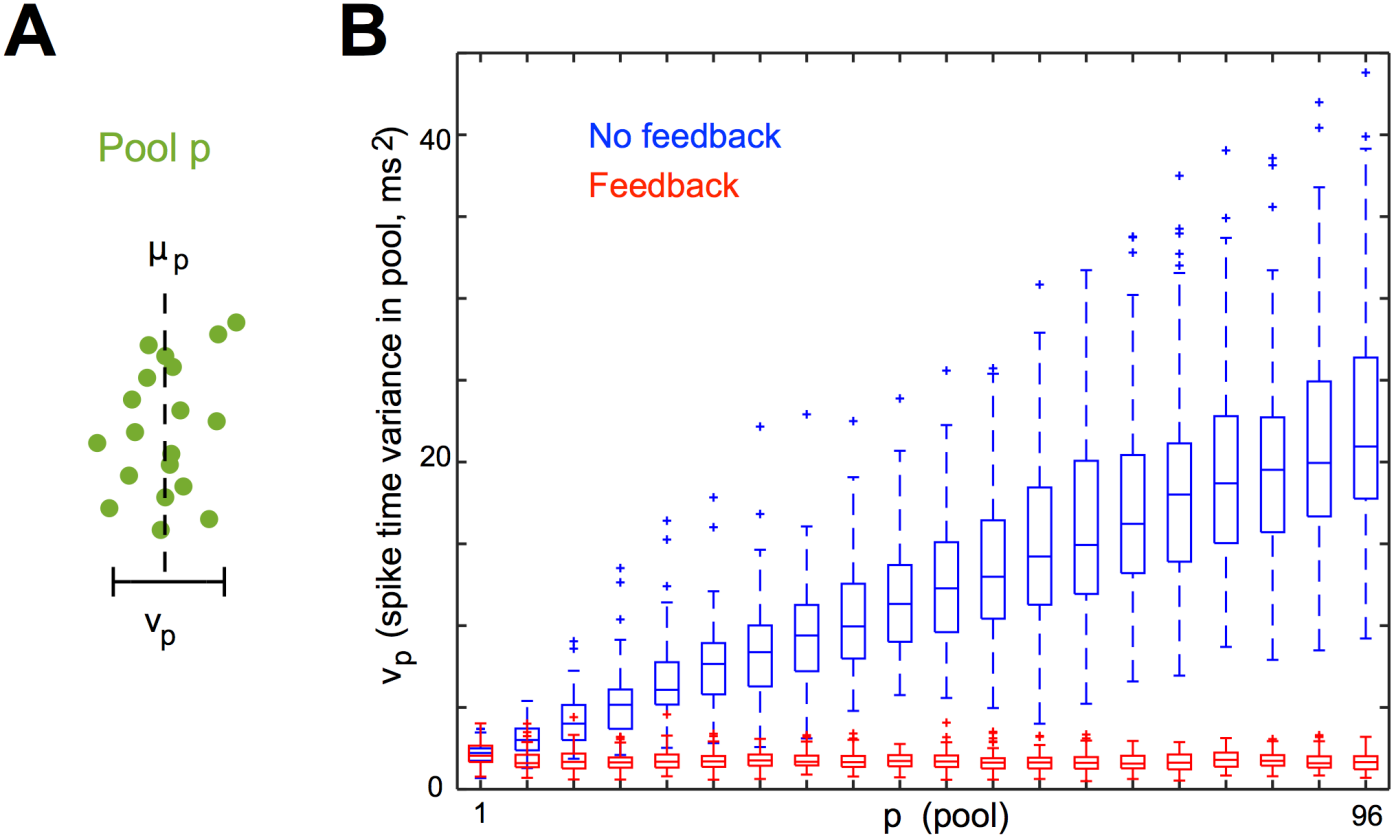
Quantification of the synchronizing effect of local feedback inhibition. **A**, Mean spike time *μ*_*p*_ and variance *v*_*p*_ are calculated for the spike times in each pool *p* in simulation 1. **B**, *v*_*p*_ is calculated for each of 100 trials for every fifth pool *p*, with and without local feedback inhibition. In both cases, each trial is initialized from a spike volley in pool 0 with *v*_0_ = 2.25. The center line of each box plot represents the median *v*_*p*_, the box spans the middle two quartiles, the whiskers span the data between q_1_-1.5(q_3_-q_1_) and q_3_+1.5(q_3_-q_1_) (where q_1_ and q_3_ are the first and third quartile, respectively), and outliers are plotted. Without feedback (blue), the variance of spike times within pool *p* increases with *p* and does not appear to converge. With feedback (red), the variance decreases slightly and appears to asymptote to a positive constant near *v*_0_.

As a measure of within-pool synchrony and its evolution over time, we calculated the mean *μ*_*p*_ and variance *v*_*p*_ of the spike times $t_{p}^{m}$ in each pool *p* for each of 100 trials of simulation 1 (Fig 6). Without feedback, the trial-averaged variance *v*_*p*_ of within-pool spike times increased without apparent bound. (This is to be expected, since the spike timing along each strand of the chain is effectively a random walk independent of the other strands.) When we introduced feedback inhibition, the trial-averaged *v*_*p*_ instead decreased slightly and appeared to stabilize. Thus, feedback prevented the progressive desynchronization of pools and instead stabilized a tight distribution of spikes about the mean.

To quantify the dependence of the synchronizing effect of local feedback on the model topology, we gradually introduced non-local E-to-I and I-to-E connections into the network in simulation 2. For each trial, we instantiated a fraction *F* of the possible global E-to-I and I-to-E connections and then normalized connection strengths to ensure that the excitatory pulse still reached the last pool. We normalized connection strengths by modifying Eq (2) to read

$$\begin{array}{cc} C_{i}dU_{n}^{m} & =\left(\frac{{\left(U_{n}^{m}\right)}^{2}}{R_{i}}+\frac{g_{ei}}{M_{e}+FPM_{e}}\sum_{p\;\text{in}\;\text{zone}\;n}\sum_{m=0}^{M_{e}-1}E\left(t-t_{p}^{m}\right)-g_{ii}\phi_{n}\right)\;dt+D_{i}dW_{n}^{m}\left(t\right)\\ d\phi_{n} & =\left(-\frac{\phi_{n}}{T_{i}}+\frac{k}{M_{i}+FM_{i}}\sum_{s}\;\delta \left(t-t_{n}^{s}\right)\right)\;dt \end{array}$$

**Fig 7:**
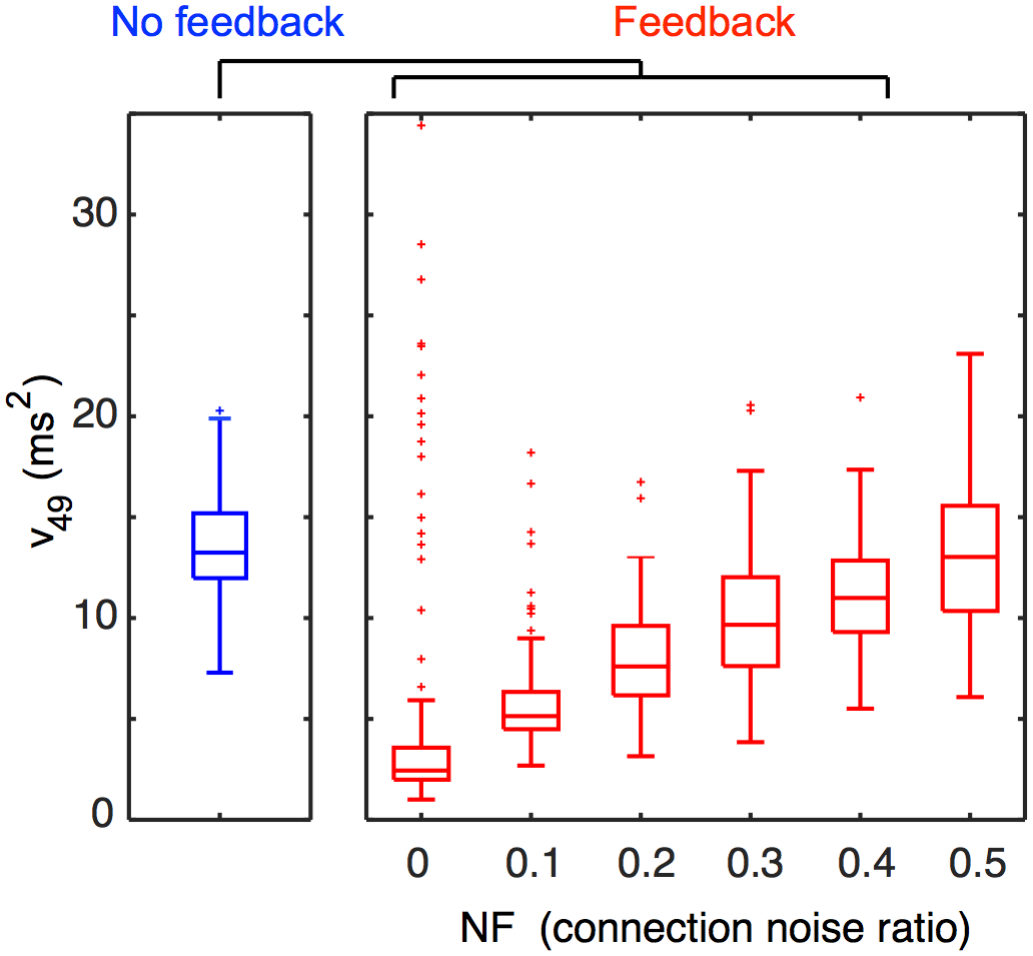
The effect of connection noise on synchronization. We tested the robustness of the synchronization effect of local inhibition against breakdown of the local network structure by introducing a fraction *F* of the possible global E-to-I and I-to-E connections into simulation 2. Since there were *N* times more possible global E-to-I (or I-to-E) than local E-to-I (or I-to-E) connections, *NF* was the ratio of global to local connections. We computed *v*_49_, the variance of spikes in pool 49, over 100 trials and for a range of values of *NF*, and made box plots as described in Fig 6. We compared the result to *v*_49_ under conditions of no feedback inhibition (blue). For *NF* ≤ 0.4, *v*_49_ was significantly different from the model with no feedback with *p* < 10^−9^ according to a Wilcoxon rank sum test with a Holm-Bonferroni correction for multiple comparisons. For *NF* = 0.5, *v*_49_ was not significantly different with or without feedback inhibition (*p* > 0.05).

The synchronizing effect of local feedback inhibition persisted until the number of added global connections reached 50% of the number of local E-to-I and I-to-E connections (Fig 7).

The synchronizing effect of local feedback inhibition on chained pools of cells can be understood as a specific instance of the more general phenomenon of synchronization by a slow-decaying pulse of shared inhibition described by Börgers and Kopell in [20]. A cell in pool *p* that receives its excitatory pulse earlier than the others in its pool also receives its pulse under a heavier blanket of inhibition, so its latency to spike is greater, whereas a cell that receives its pulse late can fire with reduced latency. Thus, decaying inhibition reigns in outlier spike times and forces spike times within a volley towards a shared mean. This intuition is explored more thoroughly in the Analysis section below.

### Improved consistency across trials

**Fig 8:**
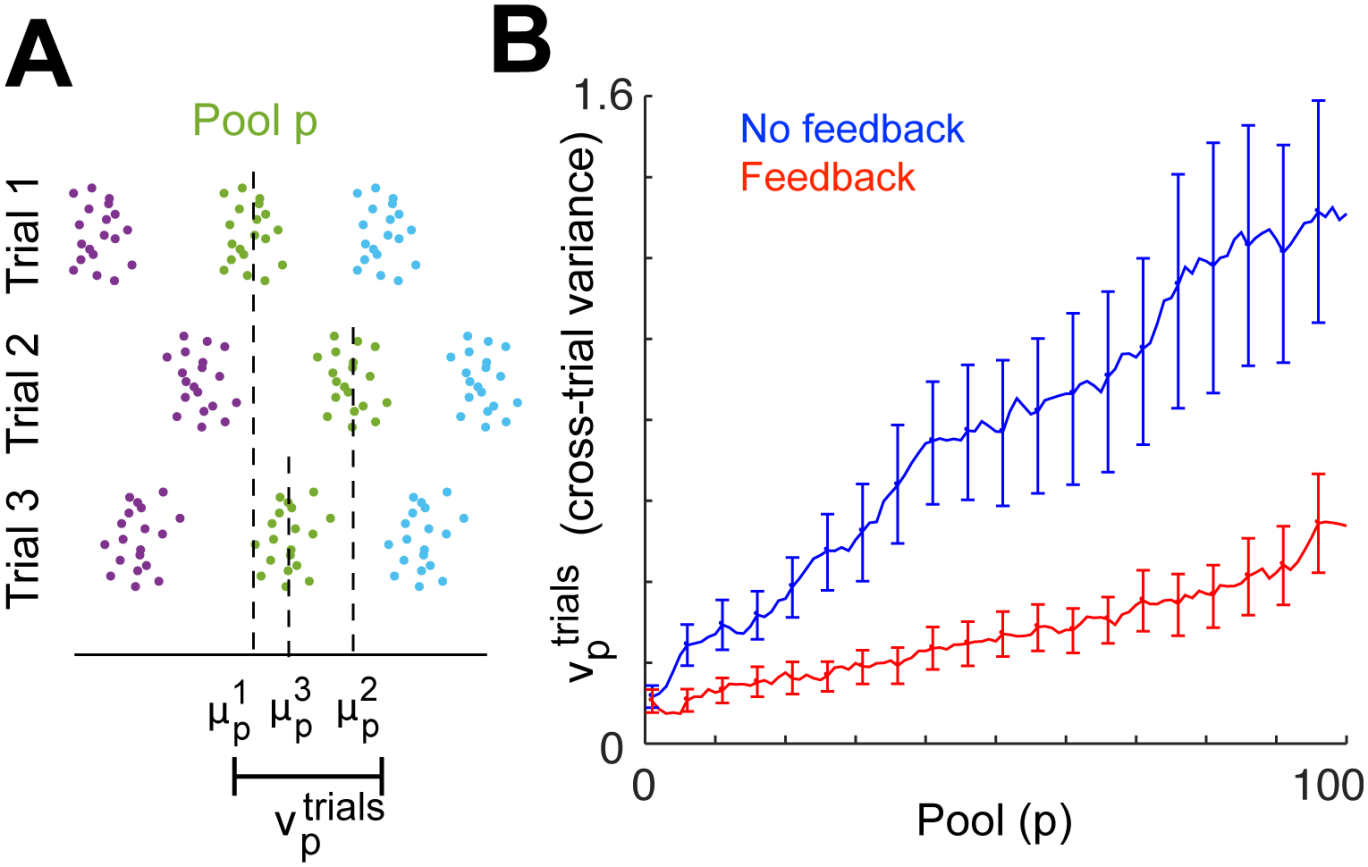
Quantification of spike volley timing variance across trials. Results are shown for simulation 1. **A**, On each simulation trial *i*, the mean spike time $\mu_{p}^{i}$ is calculated for each pool *p*, and $v_{p}^{trials}$ is defined as the variance of $\mu_{p}^{i}$ across 100 trials. **B**, Cross-trial variance $v_{p}^{trials}$ is plotted against *p*, and error bars are plotted representing 5%–95% confidence intervals calculated by case resampling. With and without local feedback inhibition, $v_{p}^{trials}$ increases roughly linearly with *p*; however, its slope is significantly shallower when local feedback inhibition is introduced.

Observations of spike rasters across 100 trials of simulation 1 suggested that in addition to synchronizing local spike volleys, local feedback inhibition also made volley timing more consistent across trials. For each simulation trial *i* and each pool *p*, we calculated the mean spike time $\mu_{p}^{i}$, and then took the variance $v_{p}^{trials}$ of these means across trials and plotted it against *p* (Fig 8).

Both with and without feedback, $v_{p}^{trials}$ increased roughly linearly. However, we found that when pools were given local feedback inhibition, the rate of increase of $v_{p}^{trials}$ was reduced. This finding is similar to the observation in [21] that, as a pulse in a synfire chain reaches a state of steady near-synchronous propagation, its propagation velocity shows less inter-trial variability. However, there are important differences between our result and theirs. In particular, they found that as the pulse approached its steady state, the time between successive spike volleys became more regular. In our data, feedback inhibition did not strongly affect the regularity of the time between the firing of pool *p* and pool *p* + 1, but had a much more noticeable effect on the regularity of the time between the firing of pool *p* and the next pool in the same zone (pool *p* + *N*). (In simulation 1, feedback decreased the cross-trial variance of the interval between mean spike times in pool 99 and pool 100 by 6.5%, as compared to a 28% decrease in variance of the interval between pools 95 and 100.) Thus, the reduction in inter-trial variability created by feedback inhibition was due to the stabilizing effect of lingering local feedback inhibition described above, which most directly influenced not the time of pulse propagation along excitatory connections, but rather the time for the pulse to circle the network and return.

### Analysis

In order to more fully understand the cause of progressive synchronization in our model with feedback, we introduced additional assumptions and approximations to make the model analytically tractable. These assumptions and approximations made it possible to linearize the dynamics around a set of excitatory spike times and allowed us to express the relationship between model parameters and the stabilization of synchrony in terms of the solution to a first passage time problem. Combining this analysis with a computational investigation of the first passage problem, we found that we could quantitatively describe the effects of IPSC decay rate, EPSC rise rate, and noise on pool synchronization. Simulation 1 met our additional assumptions, and we found that the synchronizing behavior of simulation 1 indeed agreed with our analytical predictions. Our analysis demonstrates that progressive synchronization by feedback inhibition is not a special property of a finely tuned computational model but a generic property of spatially recurrent feedforward chains with local feedback inhibition.

#### Analysis assumptions

We first assume that principal cell spike volleys are not interrupted by the firing of local inhibitory cells. In simulation 1, this condition was met because parameters were set to ensure a sufficient delay between an excitatory spike volley and the local inhibitory response. If such interruptions do not occur, we can assume that *ϕ*_*p* mod *N*_ continues to decay exponentially while the cells in pool *p* fire.

We make the approximation that, as the drive to any excitatory cell *m* in pool *p* crosses above zero, its excitatory current (described by $g_{ee}E\left(t-t_{p-1}^{m}\right)$) is well-approximated by a linearization with positive slope *a*_*p*_, where *a*_*min*_ < *a*_*p*_ < *a*_*max*_ for some constants *a*_*min*_, *a*_*max*_ > 0, and its inhibitory current (described by *g*_*ie*_ *ϕ*_*p* mod *N*_) is well-approximated by a linearization with negative slope −*b*_*p*_, where *b*_*p*_ > *b*_*min*_ for some constant *b*_*min*_ > 0 (or, in simulation 1 without feedback, *b*_*min*_ = 0). Note that the slopes *a*_*p*_ and *b*_*p*_ are assumed to be the same throughout the firing of a given pool. Let $T_{p}^{m}$ denote the first time *t* that

$$g_{ee}E\left(t_{p}^{m}-t_{p-1}^{m}\right)-g_{ie}\phi_{p\text{ mod }N}+I_{E}=0,$$ (4)

i.e., the first time that the drive to excitatory cell *m* in pool *p* crosses zero. Our approximations can be expressed as follows: for any *p*, $g_{ee}E^{\prime }\left(t-t_{p-1}^{m}\right)=a_{p}$ and $g_{ie}\phi_{p\;\text{mod}\;N}^{\prime }\left(T_{p}^{m}\right)=-b_{p}$ (where $\phi_{n}^{\prime }\left(t\right)$ denotes a temporal derivative of *ϕ*_*n*_) for all *m* = 0, …, *M*_*e*_. These approximations are most accurate while pools of cells remain near synchrony, as was the case throughout simulation 1 with feedback inhibition.

Since inhibition decays at a rate proportionate to its level, the requirement that the IPSC slope −*b*_*p*_ be negative and bounded away from zero was fulfilled in simulation 1 because regular inhibitory spiking kept *ϕ*_*n*_ bounded away from zero. The requirement that the EPSC slope be positive was fulfilled because EPSCs were large enough that the drive to any cell crossed zero during the rise of its EPSC.

Given these approximations, we can describe the EPSC to each excitatory cell in pool *p* as its drive crosses zero with the linearization:

$$g_{ee}E\left(t-t_{p-1}^{m}\right)\approx a_{p}\left(t-t_{p-1}^{m}\right)+\alpha_{p}$$ (5)

where *α*_*p*_ is a constant. Similarly, we can describe the local IPSC as the drive to cells in pool *p* crosses zero with the linearization:

$$g_{ie}\phi_{p\;\text{mod}\;N}^{\prime }\left(t\right)\approx -b_{p}t+\beta_{p}$$ (6)

where *β*_*p*_ is a constant.

Given the trajectory of *ϕ*_*p* mod *N*_ and the excitatory cell spike times $t_{p-1}^{m}$ in the upstream pool, the distribution of $t_{p}^{m}$ is determined by the solution to a first passage time problem: namely, the first passage of $V_{p}^{m}$ past *V*_*S*_, where $V_{p}^{m}$ is initialized at the beginning of the simulation. Our final assumption is that if the drive *I*(*t*) to the QIF neuron described in Eq (1) crosses zero smoothly at some time *T*, the distribution of its first spike time about *T* can be expressed as a function of only the rate *I*′(*T*) at which the neuron’s drive crosses zero (and the parameters of the QIF neuron). This assumption implies that the distribution of first spike times does not depend on initial voltage or on the drive *I*(*t*) outside a small neighborhood of the time it is brought past threshold. Under this assumption, the standard deviation of the distribution of first spike times can be expressed as *σ*(*I*′(*T*)). In S1A and S1B Fig, we show computational results demonstrating that the first passage time distribution of a QIF neuron as its drive crosses threshold is indeed insensitive to the initial voltage and to the time course of *I*(*t*) outside a small neighborhood of *T* within the regimes relevant to this system. We also show the dependence of this distribution on threshold-crossing rate (S1C Fig), and plot *σ*(*I*′(*T*)) (S1D Fig), noting that the standard deviation of the first passage time decreases with increasing *I*′(*T*).

In S1 Text, we use this assumption to show that $t_{p}^{m}=T_{p}^{m}+z_{p}+\xi_{p}^{m}$, where $T_{p}^{m}$ is the time excitation crosses inhibition in cell *m* in pool *p*, *z*_*p*_ is a constant for each pool *p*, and $\xi_{p}^{m}$ is a random variable drawn from a pool-specific mean-zero distribution with standard deviation *σ*_*p*_.

#### Analysis results

We want to describe the evolution of intra-pool synchrony over time. We define *μ*_*p*_ to be the mean firing time in pool *p*, and we consider the variance *v*_*p*_ of spike times within each pool *p*:

$$\mu_{p}≔\frac{1}{M_{e}}\sum_{m=0}^{M_{e}-1}t_{p}^{m}$$ (7)

$$v_{p}≔\frac{1}{M_{e}-1}\sum_{m=0}^{M_{e}-1}{\left(t_{p}^{m}-\mu_{p}\right)}^{2},$$ (8)

The variance *v*_*p*_ is a measure of synchrony of spiking in pool *p*. If *v*_*p*_ = 0, synchrony is perfect; larger *v*_*p*_ denotes a higher degree of asynchrony. $E[v_{p}]$, the expected value of the variance in pool *p* given initial conditions, is a measure of the expected degree of synchrony in pool *p*.

In S1 Text, we find a recursive relation for $E[v_{p}]$ that allows us to calculate the expected variance within pool *p* in terms of $E[v_{p-1}]$:

$$E\left[v_{p}\right]={\left(\frac{a_{p}}{a_{p}+b_{p}}\right)}^{2}E[v_{p-1}]+\sigma_{p}^{2}.$$ (9)

In our simulations without feedback, *b*_*p*_ = 0, so this expression predicts that expected variance should grow linearly at rate $\sigma_{p}^{2}=\sigma {\left(a_{p}\right)}^{2}$ per pool.

In S1 Text, we demonstrate that when *b*_*p*_ > 0, the recursive relation sets an asymptotic upper bound on $E[v_{p}]$:

$$\lim_{p\to \infty }E\left[v_{p}\right]<\frac{\sigma {\left(a_{min}+b_{min}\right)}^{2}}{1-{\left(\frac{a_{max}}{a_{max}+b_{min}}\right)}^{2}}$$ (10)

where the function *σ*(⋅) is the standard deviation of the QIF neuron’s first passage time as a decreasing function of the rate of depolarization. In other words, the expected variance of spike times within a pool eventually becomes less than $\frac{\sigma {\left(a_{min}+b_{min}\right)}^{2}}{1-{\left(\frac{a_{max}}{a_{max}+b_{min}}\right)}^{2}}$, and remain so. Since *σ*(*a*_*min* +_ *b*_*min*_) and $\frac{1}{1-{\left(\frac{a_{max}}{a_{max}+b_{min}}\right)}^{2}}$ both decrease with increasing *b*_*min*_, the asymptotic upper bound on $E[v_{p}]$ is lower if *b*_*min*_ is larger, i.e., if inhibition is decaying more sharply as local pools fire.

**Fig 9:**
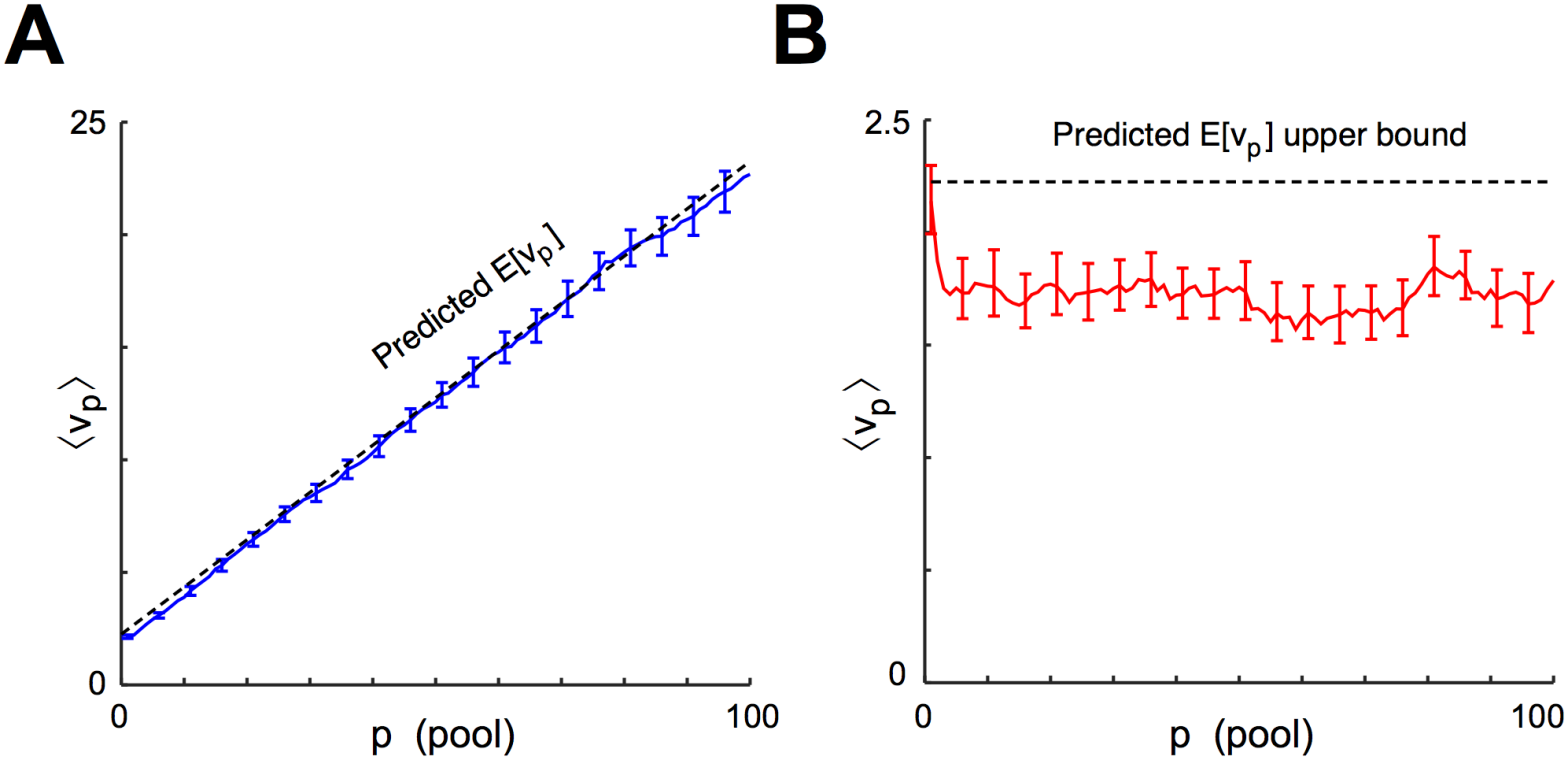
Simulation results match analysis predictions. **A**, For 100 repetitions of simulation 1 without feedback inhibition, the mean ⟨*v*_*p*_⟩ is calculated across trials and plotted against *p*, and error bars representing 10%–90% confidence intervals are calculated for every fifth pool by case resampling. The rate of increase of ⟨*v*_*p*_⟩ closely matches the theory (dotted line) except in the first few pools, where the effect of initializing all voltages from zero may have artificially decreased the observed level of synchrony. **B** For 100 repetitions of simulation 1 with feedback inhibition, ⟨*v*_*p*_⟩ is plotted with error bars representing 1%–99% confidence intervals calculated by case resampling. From pool 6 on, confidence intervals remain below the upper bound for $E[v_{p}]$ estimated by our analysis (dotted line).

We validated our analytical results by checking them against the results of simulation 1. In simulation 1 without feedback, we approximated *a*_*p*_ by measuring the slope of rising excitation over 4ms leading up to each spike, and averaging over all spikes. We then calculated *σ*(*a*_*p*_) by applying ramp depolarizations to QIF neurons as described in S1 Fig, and found that *σ*(*a*_*p*_)^2^ ≈ 0.21*ms* ^2^. In Fig 9A, the dashed black line represents the growth of within-pool variance at rate 0.21*ms* ^2^ per pool predicted by our analysis. Our prediction falls close to the cross-trial mean of *v*_*p*_, with some small deviation for early pools that may reflect a small artificial increase in synchrony due to initializing all cell voltages together.

In simulation 1 with feedback, we compared the upper bound on within-pool variance predicted by our analysis with the variance observed in simulation. Since the appropriate spatiotemporal coordination of the pulse and local inhibition is not present until the pulse has circled the network once, we considered only pools *p* ≥ 5. We computed *a*_*p*_ and *b*_*p*_ for pools *p* ≥ 5 by averaging the slope of the PSCs over the 4ms leading up to each spike, and then averaging the result over all spikes in pool *p*. We set *a*_*max*_, *a*_*min*_ and *b*_*min*_ to the maximal and minimal values of *a*_*p*_ and the minimal value of *b*_*p*_, respectively, during this run. We found that *a*_*max*_ = 0.0787, *a*_*min*_ = 0.0702, and *b*_*min*_ = 0.0042. By running QIF simulations as described in S1 Fig, we calculated *σ*(*a*_*min*_+*b*_*min*_) = 0.47. Thus, the upper bound on the variance predicted by our analytical results was

$$\lim_{p\to \infty }E\left[v_{p}\right]<\frac{\sigma {\left(a_{min}+b_{min}\right)}^{2}}{1-{\left(\frac{a_{max}}{a_{max}+b_{min}}\right)}^{2}}=\frac{0.47^{2}}{1-{\left(\frac{0.0787}{0.0787+0.0042}\right)}^{2}}=2.23.$$

In Fig 9B, we show that the mean of *v*_*p*_ over trials was less than 2.23 (dotted line) with 99% confidence for all *p* ≥ *N*, strongly suggesting that the true value of $E[v_{p}]$ was below this upper bound.

## Discussion

We have put forward a model of sequence generation based on recent experimental findings in the songbird [12, 13]. In this model, a feedforward chain of excitatory neurons passes repeatedly through multiple zones of inhibition, triggering local feedback inhibition in each. We have shown that this model can generate stereotyped neural sequences, creating synchrony among pools of cells through shared inhibition and stabilizing inter-trial spike timing. These effects can operate in place of (or, presumably, in cooperation with) the similar effects of the redundant feedforward excitatory connectivity that characterizes the synfire chain.

Though previous models of neural sequence generation have used inhibition in a variety of ways [14, 22–25], they are all fundamentally distinct in structure and dynamics from our model. In “winnerless competition” models inspired by the dynamics of insect olfaction, Rabinovich et al. [22] generate sequences through competitive inhibitory interactions in a randomly connected network. Verduzco-Flores et al. [24] and Assisi et al. [23] use a network of excitatory and inhibitory units to learn and generate sequences that propagate using a combination of disynaptic inhibition and adaptation currents. A series of modeling papers have proposed that sequences are generated using strong global (not local) inhibition to select between multiple possible synfire chains [9, 10, 14, 15, 26]. Some of these have incorporated precise spatial constraints on otherwise global inhibitory connectivity in order to disinhibit principal cells at the appropriate times [14, 15]. Like us, Gibb et al. [14] and Bertram et al. [25] explore models of feedforward chains of excitatory cells with local inhibition, but they give their excitatory chains no manner of spatial recurrence, so the local inhibition evoked by a pool of excitatory cells cannot affect the spiking of cells at a later point along the chain. Our model is unique in the use of spatially recurrent excitatory chains, and in the use of local feedback inhibition to stabilize synchrony (rather than, e.g., to propagate spiking by inducing rebounds as in [25]).

We have shown in simulation that our model of an excitatory chain spiraling through inhibitory zones creates local alternation between excitatory and inhibitory cells, consistent with the observation of cell-type specific phase preferences in the 30Hz component of the local field potential in HVC [13]. Moreover, it reproduces the observation that spiking is not globally phase-coordinated, but occurs continuously throughout song. None of the models discussed above produce such a firing pattern—it is a natural consequence of localized inhibition and spatially recurrent excitatory activity, the same factors that differentiate our model from previous work and produce its synchronizing and temporally stabilizing dynamics. Our model is also consistent with paired recordings in slice, which have shown that excitatory neurons in HVC contact each other primarily through disynaptic inhibition [12, 27] as would be expected in a network dominated by local inhibitory feedback and with only sparse, specific monosynaptic connections between principal cells. Finally, there is small but growing evidence that HVC activity is correlated over space [28, 29] and that HVC connectivity is spatially structured [12, 30–32], consistent with a model in which spatial regions of HVC act as inhibitory zones.

However, our model deviates from what is known experimentally in two important respects. First, interneurons in our model fire with strong periodicity, yet HVC interneurons have dense firing patterns with intermittent periodicity during singing [13, 33] (see S2 Fig). Additionally, the stereotyped ≈ 30 Hz LFP, which is correlated with interneuron firing, is not a perfectly periodic signal [13]. The periodicity in the model is a result of its highly simplified structure. If the topological structure of the chain were more complex than a simple spiral, inhibitory activity might more closely resemble what has been observed experimentally; however, this is beyond the scope of the current study. Second, our model implies that inhibitory interactions between HVC_RA_ neurons should be primarily localized to a subregion of HVC, but recent evidence suggests that HVC_RA_ pairs inhibit each other over relatively large distances (hundreds of *μ*m) [12]. Our simulations with added global connectivity (S2 Fig) show that our model is robust to between-zone disynaptic inhibition up to a 2:1 local-to-global connection ratio. Moreover, future experiments will be needed to directly observe whether disynaptic inhibition between HVC_RA_ neurons is in fact local or global.

As constructed for this study, our model has the capacity to play back only one sequence. In the case of the zebra finch, which learns only one song, this is an appropriate constraint, but this limitation would have to be addressed for broader applications. Storage of and selection between multiple sequences has been explored by other authors, both in HVC [9, 14, 15] and more generally [10, 26, 34, 35]. We note that the spatial recurrence which is central to our model could be exploited for this purpose: multiple disconnected chains passing through the same sequence of regions could activate the same cycle of local feedback inhibition and benefit from the same stabilization of timing.

We have demonstrated in simulation and through proof that the presence of shared decaying inhibition progressively synchronizes the firing of pools of excitatory cells. We have also derived a specific asymptotic upper bound for the expected variance that decreases with increasing *b*_*min*_, where *b*_*min*_ represents an upper bound on the magnitude of the decay rate of inhibition during local excitatory spiking. The more sharply the local inhibition is decaying during the spiking of a pool, the larger a value we can choose for *b*_*min*_. Consequently, the more sharply local feedback inhibition is decaying when a pool of cells spikes, the tighter the resulting synchrony guaranteed by our analysis. (We note that there is a non-trivial relationship between the exponential time constant *T*_*i*_ of inhibitory decay and the instantaneous rate of inhibitory decay—since inhibitory decay is exponential, the latter also depends on the level of inhibition and thus on the recent inhibitory spiking history.)

We have also shown in simulation that local feedback inhibition creates sequences with timing that is more stereotyped across trials. One possible intuitive explanation for this effect is that the inhibitory state of each zone stores information about the timing of the most recent local volley; thus, the drift in volley timing due to noise can be partially corrected as the excitatory pulse reaches each zone. In other words, the information about the timing of the previous volley delivered by E-to-E connections and the information about the timing of the most recent local volley delivered by I-to-E connections are both incorporated to determine the timing of each spike volley.

Our model is closely related to the mechanism of “communication through resonance” developed by Hahn et al. [36]. In their model, pools of cells are synchronized by cycles of inhibition evoked by the arrival of external periodic excitatory pulses, whereas in our model pools of cells are synchronized by cycles of inhibition evoked by a single excitatory pulse as it returns periodically to each inhibitory zone. A similar model developed by Jahnke et al. [37] supports synchronous propagation of pulses through a network by imposing global oscillations resonant with transmission delays. Their model requires net-excitatory oscillating input or nonlinear coupling in order to keep the pulse from dying while the network is inhibited. In our model, these global oscillations are replaced by multiple local oscillations at different phases. Although the oscillations involve periods of inhibition, our network is never globally inhibited, circumventing this possible cause of propagation failure.

It is important to note the relationship between our model and the ideas discussed by Long et al. in [2]. They show that principal cells in HVC routinely receive strong depolarizations immediately before spiking. They use this observation to support an excitatory chain model and reject a model in which cells fire as a ramp of excitatory drive pushes them sequentially past their firing thresholds. Our model effectively combines these two mechanisms: cells receive strong depolarizations due to the excitatory chain structure embedded in the network, but the timing of spikes is also influenced by more gradual downward ramps of inhibition that affect (but do not fully determine) the time that the drive to each cell crosses threshold. Since the timing of sequential activity is influenced by the local network state as well as the arrival of an excitatory pulse, a model of this type would be better suited than a synfire chain to produce sequential activity that is non-uniform in time [38].

It could be argued that a chain of excitatory connections with periodic spatial recurrence is biophysically implausible. However, our model only requires that excitatory activity pass through inhibitory zones sequentially and return to them regularly; preliminary simulations suggest that such a pattern of activity may be achievable on a random excitatory network grouped into zones with localized inhibitory feedback. As an excitatory pulse propagates through the network, it is followed by a wave of local inhibition. This inhibition prevents excitation from returning to a zone until it has decayed sufficiently. Once it has decayed, random percolation ensures that activity does return. The result is a pulse that passes through the zones in an order determined by connectivity and initial levels of inhibition, and that meets the decaying inhibition from its previous pass as it reaches each zone. This pulse may activate different cells on each pass, generating a sequence significantly longer than a single pass through the network. The extraneous excitatory connections in such a random network would trigger EPSCs in some cells while they remained under strong inhibition, potentially explaining the observation of multiple stereotyped depolarizations in HVC_RA_ cells during zebra finch song [12]. Furthermore, as regions are repeatedly activated in the same order, the network might use spike-timing-dependent plasticity to learn the excitatory connections necessary to reliably reproduce this pattern, ultimately creating the circulating chain architecture assumed in our model. In Fig 7, we show that synchronization through local feedback is somewhat robust to connection noise—once network connectivity became sufficiently localized, feedback inhibition would begin to contribute to pulse synchronization.

For this mechanism to function, excitatory activity returning to a region of HVC must arrive during the decaying slope of the inhibition, and must therefore circulate through the inhibitory zones on a time scale matching the decay of inhibition. In our model, this is achieved through manual tuning. However, a learning process in HVC like the one described above might help to match these timescales by ensuring that excitatory activity returns to a zone as soon as inhibition decays sufficiently to permit it. Alternatively, the timescale of recurrence may relate to the cortical-thalamic loop cycle time, which does appear to match the time-constant of inhibitory decay [12]. In this view, the spiral does not exist entirely in HVC, but instead passes through the cortical-thalamic loop.

Our model also may throw a new light on certain dynamics in the mammalian brain. Two brain regions in which precisely-timed sequential activity is thought to be essential are the motor cortex [39] and hippocampus [40]. Both of these regions have been shown to support rhythmic traveling waves, at beta frequencies (15–30Hz) [41] and theta frequencies (4–10Hz) [42] respectively. We suggest that these waves may be a manifestation of the locally-coordinated, globally out-of-phase inhibitory cycles that characterize our model and help synchronize neuronal pools and stabilize timing within a firing sequence.

## Supporting Information

S1 Fig The effects of parameters on QIF neuron first-passage time. Parameters for the QIF neuron are those chosen for the excitatory neurons in the model (see Table 1). The QIF neuron is depolarized by a current ramp *I*(*t*) that crosses zero at time *T* = 0. Simulations are performed varying the steepness *I*′(*T*) of the current ramp, its beginning and ending height, and the initial QIF voltage *V*_0_ within the approximate range relevant to QIF neurons in our simulation. The first passage time distribution of the neuron changes with *I*′(*T*); however, within the regime relevant to the model presented here, first passage time distribution is insensitive to the initial voltage *V*_0_ and to the starting and ending point of the current ramp. Intuitively, this is the case because these neurons lack long time scale terms, so in noisy conditions they have very limited memory of recent state and input history. Below, the mean of the distribution of first passage times is plotted as conditions are varied. Shading indicates standard deviation. Above, depolarizing ramps are plotted for several values of each parameter. **A**, The initial voltage of the neuron is varied. Unless the initial voltage is very close to threshold, this does not significantly affect the first passage time distribution. **B**, The depolarizing ramp is shifted diagonally such that the initial and final drive vary but the depolarizing ramp crosses threshold at the same rate and time. Unless the initial drive is close to threshold or the final drive does not cross threshold, this does not significantly affect the first passage time distribution. **C**, The rate of depolarization is varied. Increasing the steepness of the current ramp slightly decreases the mean first passage time and tightens the distribution. **D**, The standard deviation *σ* of the first passage time is plotted as a function of depolarization rate *I*′(*T*). Note that *σ* decreases with increasing *I*′(*T*).
(TIFF)

S2 Fig Observed interneuron spike raster over multiple trials. Data from [11]. Trials are stacked vertically and shifted in time to best match song features with a template (no time warping was applied). Individual interneurons in HVC produce spike trains that are highly stereotyped over trials. Spike trains are not periodic, but show windows of apparent periodicity.
(TIFF)

S1 Text Derivation of recurrence relation and upper bound for expected spike time variance. This text contains a derivation of Eq (9), and demonstrates that this recurrence implies asymptotic upper bound Eq (10) on expected spike time variance, which decreases with *b*_*min*_.
(PDF)
