# Supplementary figures and images for: Neural Sequence Generation Using Spatiotemporal Patterns of Inhibition
Source: PLoS Comput Biol. 2015 Nov 4;11(11):e1004581. doi: 10.1371/journal.pcbi.1004581 (PMC4633124; doi:10.1371/journal.pcbi.1004581)

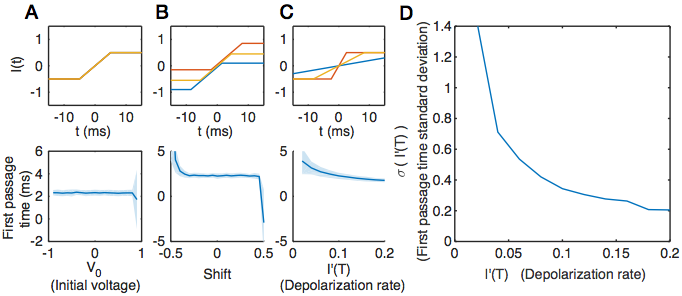

Supplement: S1 Fig — Parameters for the QIF neuron are those chosen for the excitatory neurons in the model (see Table 1). The QIF neuron is depolarized by a current ramp I(t) that crosses zero at time T = 0. Simulations are performed varying the steepness I′(T) of the current ramp, its beginning and ending height, and the initial QIF voltage V 0 within the approximate range relevant to QIF neurons in our simulation. The first passage time distribution of the neuron changes with I′(T); however, within the regime relevant to the model presented here, first passage time distribution is insensitive to the initial voltage V 0 and to the starting and ending point of the current ramp. Intuitively, this is the case because these neurons lack long time scale terms, so in noisy conditions they have very limited memory of recent state and input history. Below, the mean of the distribution of first passage times is plotted as conditions are varied. Shading indicates standard deviation. Above, depolarizing ramps are plotted for several values of each parameter. A, The initial voltage of the neuron is varied. Unless the initial voltage is very close to threshold, this does not significantly affect the first passage time distribution. B, The depolarizing ramp is shifted diagonally such that the initial and final drive vary but the depolarizing ramp crosses threshold at the same rate and time. Unless the initial drive is close to threshold or the final drive does not cross threshold, this does not significantly affect the first passage time distribution. C, The rate of depolarization is varied. Increasing the steepness of the current ramp slightly decreases the mean first passage time and tightens the distribution. D, The standard deviation σ of the first passage time is plotted as a function of depolarization rate I′(T). Note that σ decreases with increasing I′(T). (TIFF) [file pcbi.1004581.s001.tiff]

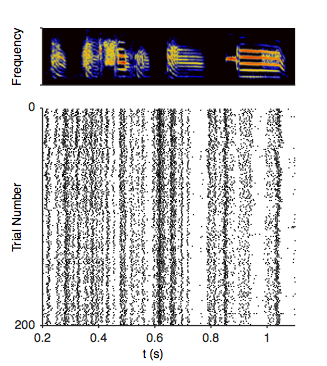

Supplement: S2 Fig — Data from [11]. Trials are stacked vertically and shifted in time to best match song features with a template (no time warping was applied). Individual interneurons in HVC produce spike trains that are highly stereotyped over trials. Spike trains are not periodic, but show windows of apparent periodicity. (TIFF) [file pcbi.1004581.s002.tiff]
