# Supplementary material for: Neural Sequence Generation Using Spatiotemporal Patterns of Inhibition
Source: PLoS Comput Biol. 2015 Nov 4;11(11):e1004581. doi: 10.1371/journal.pcbi.1004581 (PMC4633124; doi:10.1371/journal.pcbi.1004581)
Supplement: S1 Text — This text contains a derivation of Eq (9), and demonstrates that this recurrence implies asymptotic upper bound Eq (10) on expected spike time variance, which decreases with b min. (PDF) [file pcbi.1004581.s003.pdf]

Neural sequence generation using spatiotemporal patterns of inhibition: S1 Text. Derivation of recurrence relation and upper bound for expected spike time variance.

In this text we derive the recurrence relation for the expected variance of spike times in pool p used in the main manuscript, and show that this recurrence relation implies an asymptotic upper bound on expected spike time variance that decreases as b_{min} increases. In order to derive the recursion, we first derive an expression for the random variable t_p^m; we use this expression to write a recursive formula for t_p^m; we use this formula to derive a recursive formula for mean spike time within pools; and finally, we use both formulas to derive the recursive formula for expected variance.

Expression for spike time in terms of pool-specific variables

We assume for the sake of analysis that the first passage time of a QIF neuron depolarized past zero at time T is drawn from a distribution that depends only on the rate of depolarization $I'(T)$ (and the QIF neuron parameters). We justify this assumption with simulation results in Fig. S1. Intuitively, this is the case because these model neurons have no long time scales, and therefore quickly lose any memory of initial voltage state and recent input history as long as they remain below threshold.

Given this assumption, we can let $\rho\,(t-T; I'(T))$ denote the distribution of any excitatory cell's spike time about T (the time its drive $I(t)$ crosses zero), as a function of $I'(T)$. From equations (5) and (6) we find that the drive $I(t) = g_{ee}E(t - t_{p-1}^m) - g_{ie}\phi_{p \text{ mod } N} + I_E$ to principal cell m in pool p crosses zero at the same rate for all m:

$$I'(T_p^m) = g_{ee}E'(T_p^m - t_{p-1}^m) - g_{ie}\phi'_{p \text{ mod } N}(t) \approx a_p + b_p.$$

Therefore, the spike times of all neurons in pool p are drawn from identical distributions relative to the time each one's drive crosses zero: spike time t_p^m is drawn from the distribution $\rho(t - T_p^m; a_p + b_p)$ for all m.

We can express t_p^m as a sum of an expected value and a mean-zero random variable. Let $z(I'(T))$ denote the mean of $\rho(t - T; I'(T))$ and let $\sigma(I'(T))$ denote its standard deviation, both functions of $I'(T)$. We define

$$\begin{aligned} z_p :=& z(a_p + b_p) \\ \sigma_p :=& \sigma(a_p + b_p). \end{aligned} \tag{11}$$

Given a_p and b_p, the expected value of t_p^m is $T_p^m + z_p$, and we can express the random variable t_p^m as

$$t_p^m = T_p^m + z_p + \xi_p^m \tag{12}$$

where ξ_p^m is a random variable drawn from the mean-zero distribution $\rho(t - T_p^m + z_p; a_p + b_p)$ with variance σ_p.

Recursive formula for spike time

First we solve for a spike time t_p^m in terms of t_{p-1}^m (the previous spike time on strand m). Substituting (5) and (6) into (4), we have

$$\begin{aligned} 0 =& a_p(T_p^m - t_{p-1}^m) + \alpha_p + b_pT_p^m - \beta_p + I_E \\ T_p^m =& \frac{1}{a_p + b_p}\left(\beta_p - \alpha_p - I_E + a_pt_{p-1}^m\right) \end{aligned}$$

Substituting into (12),

$$t_p^m = \frac{1}{a_p + b_p}\left(\beta_p - \alpha_p - I_E + a_p t_{p-1}^m\right) + \xi_p^m + z_p \tag{13}$$

This formula gives the spike time of cell m in pool p as a function of t_{p-1}^m (the spike time of the upstream cell, cell m in pool $p-1$).

Recursive formula for mean spike time

As in (7), we define μ_p to be the mean spike time in pool p:

$$\mu_p := \frac{1}{M_e}\sum_{\tilde{m}=0}^{M_e-1} t_p^{\tilde{m}}. \tag{14}$$

Next we write a recursive relation for μ_p, the mean spike time in pool p, in terms off μ_{p-1} (the mean spike time in the upstream pool).

Substituting (13) into (14),

$$\begin{aligned}\mu_p =& \frac{1}{M_e}\sum_{\tilde{m}=0}^{M_e-1}\left(\frac{1}{a_p + b_p}\left(\beta_p - \alpha_p - I_E + a_p t_{p-1}^{\tilde{m}}\right) + \xi_p^{\tilde{m}} + z_p\right)\\ =& \frac{1}{a_p + b_p}\left(\beta_p - \alpha_p - I_E + a_p\left(\frac{1}{M_e}\sum_{\tilde{m}=0}^{M_e-1} t_{p-1}^{\tilde{m}}\right)\right) + z_p + \frac{1}{M_e}\sum_{\tilde{m}=0}^{M_e-1}\xi_p^{\tilde{m}}\end{aligned}$$

Now we can substitute from (14) and write

$$\mu_p = \frac{1}{a_p + b_p}\left(\beta_p - \alpha_p - I_E + a_p\mu_{p-1}\right) + z_p + \frac{1}{M_e}\sum_{\tilde{m}=0}^{M_e-1}\xi_p^{\tilde{m}} \tag{15}$$

This is an expression for mean spike time in pool p in terms of the most recent mean spike time.

Recursive formula for expected variance

As in (8), we define v_p to be the variance among the spike times in pool p:

$$v_p := \frac{1}{M_e - 1}\sum_{m=0}^{M_e-1}(t_p^m - \mu_p)^2. \tag{16}$$

Here we derive a recursive formula for the expected variance of spike times within a pool, $\mathbb{E}[v_p^m]$, in terms of the expected variance of the preceding pool, $\mathbb{E}[v_{p-1}^m]$.

First, we write an expression for $t_p^m - \mu_p$, the difference between a single spike time in pool p and the mean spike time in that pool. From (13) and (15), we have

$$\begin{aligned}t_p^m - \mu_p =& \frac{1}{a_p + b_p}\left(\beta_p - \alpha_p + I_E + a_p t_{p-1}^m\right) + \xi_p^m + z_p \ldots\\ &- \left(\frac{1}{a_p + b_p}\left(\beta_p - \alpha_p + I_E + a_p\mu_{p-1}\right) + z_p + \frac{1}{M_e}\sum_{\tilde{m}=0}^{M_e-1}\xi_p^{\tilde{m}}\right)\end{aligned}$$

The constants cancel, leaving

$$=\frac{a_p}{a_p+b_p}\left(t_{p-1}^m-\mu_{p-1}\right)+\xi_p^m-\frac{1}{M_e}\sum_{\tilde{m}=0}^{M_e-1}\xi_p^{\tilde{m}}$$

Combining the two ξ_p^m terms,

$$=\frac{a_p}{a_p+b_p}\left(t_{p-1}^m-\mu_{p-1}\right)+\frac{M_e-1}{M_e}\xi_p^m-\frac{1}{M_e}\sum_{\tilde{m}\neq m}\xi_p^{\tilde{m}} \tag{17}$$

Next, we solve for the expected value of the variance in pool p in terms of the expected variance in pool $p-1$. From (16), we have

$$\mathbb{E}[v_p]=\mathbb{E}\left[\frac{1}{M_e-1}\sum_{m=0}^{M_e-1}\left(t_p^m-\mu_p\right)^2\right]$$

Substituting for $t_p^m-\mu_p$ from (17),

$$\mathbb{E}[v_p]=\mathbb{E}\left[\frac{1}{M_e-1}\sum_{m=0}^{M_e-1}\left(\frac{a_p}{a_p+b_p}\left(t_{p-1}^m-\mu_{p-1}\right)+\frac{M_e-1}{M_e}\xi_p^m-\frac{1}{M_e}\sum_{\tilde{m}\neq m}\xi_p^{\tilde{m}}\right)^2\right]$$

When this expression is squared through, cross terms of the form $k\xi_p^m$ can be eliminated since ξ_p^m has zero mean, and cross terms of the form $k\xi_p^m\xi_p^{\tilde{m}}$ for $\tilde{m}\neq m$ can be eliminated by the independence of these variables. We are left with

$$\begin{aligned}
\mathbb{E}[v_p]=&\mathbb{E}\left[\frac{1}{M_e-1}\sum_{m=0}^{M_e-1}\left(\left(\frac{a_p}{a_p+b_p}\left(t_{p-1}^m-\mu_{p-1}\right)\right)^2+\left(\frac{M_e-1}{M_e}\right)^2\left(\xi_p^m\right)^2+\frac{1}{M_e^2}\sum_{\tilde{m}\neq m}\left(\xi_p^{\tilde{m}}\right)^2\right)\right]\\
=&\mathbb{E}\left[\frac{1}{M_e-1}\sum_{m=0}^{M_e-1}\left(\frac{a_p}{a_p+b_p}\left(t_{p-1}^m-\mu_{p-1}\right)\right)^2\right]+\mathbb{E}\left[\frac{1}{M_e-1}\sum_{m=0}^{M_e-1}\left(\frac{M_e-1}{M_e}\right)^2\left(\xi_p^m\right)^2\right]\dots\\
&+\mathbb{E}\left[\frac{1}{M_e-1}\sum_{m=0}^{M_e-1}\frac{1}{M_e^2}\sum_{\tilde{m}\neq m}\left(\xi_p^{\tilde{m}}\right)^2\right]\\
=&\left(\frac{a_p}{a_p+b_p}\right)^2\mathbb{E}\left[\frac{1}{M_e-1}\sum_{m=0}^{M_e-1}\left(t_{p-1}^m-\mu_{p-1}\right)^2\right]+\frac{1}{M_e-1}\left(\frac{M_e-1}{M_e}\right)^2\sum_{m=0}^{M_e-1}\mathbb{E}\left[\left(\xi_p^m\right)^2\right]\dots\\
&+\frac{1}{M_e^2(M_e-1)}\sum_{m=0}^{M_e-1}\sum_{\tilde{m}\neq m}\mathbb{E}\left[\left(\xi_p^{\tilde{m}}\right)^2\right]
\end{aligned}$$

Substituting from (16) and using $\mathbb{E}\left[(\xi_p^m)^2\right] = \sigma_p^2$,

$$\begin{aligned}
\mathbb{E}[v_p] &= \left(\frac{a_p}{a_p+b_p}\right)^2 \mathbb{E}\left[v_{p-1}\right] + \frac{1}{M_e-1}\left(\frac{M_e-1}{M_e}\right)^2 \sum_{m=0}^{M_e-1} \sigma_p^2 + \frac{1}{M_e^2(M_e-1)} \sum_{m=0}^{M_e-1} \sum_{\tilde{m}\neq m} \sigma_p^2 \\
&= \left(\frac{a_p}{a_p+b_p}\right)^2 \mathbb{E}\left[v_{p-1}\right] + \frac{1}{M_e-1}\left(\frac{M_e-1}{M_e}\right)^2 M_e\sigma_p^2 + \frac{1}{M_e^2(M_e-1)} M_e(M_e-1)\sigma_p^2 \\
&= \left(\frac{a_p}{a_p+b_p}\right)^2 \mathbb{E}\left[v_{p-1}\right] + \frac{M_e-1}{M_e}\sigma_p^2 + \frac{1}{M_e}\sigma_p^2 \\
&= \left(\frac{a_p}{a_p+b_p}\right)^2 \mathbb{E}\left[v_{p-1}\right] + \sigma_p^2. \qquad (18)
\end{aligned}$$

Note that if $b_p = 0$, this formula predicts that $\mathbb{E}\left[v_p\right]$ will grow linearly at a rate of $\sigma_p^2 = \sigma(a_p)^2$ per pool.

Asymptotic bound on expected variance

Here we show that, when inhibitory feedback is nonzero, the recursive expression for $\mathbb{E}[v_p]$ derived above implies an asymptotic upper bound $\frac{\sigma(a_{min}+b_{min})}{1-\left(\frac{a_{max}}{a_{max}+b_{min}}\right)^2}$ on expected variance, where $\sigma(a_{min}+b_{min})$ decreases with increasing b_{min}.

By assumption, there exist positive constants a_{max}, a_{min} and b_{min} such that $b_{min} \leq b_p$ and $a_{min} \leq a_p \leq a_{max}$, so $\frac{a_p}{a_p+b_p} \leq \frac{a_{max}}{a_{max}+b_{min}}$. Substituting this inequality into (18),

$$\mathbb{E}[v_p] \leq \left(\frac{a_{max}}{a_{max}+b_{min}}\right)^2 \mathbb{E}\left[v_{p-1}\right] + \sigma_p^2.$$

We recall from equation (11) that $\sigma_p := \sigma(a_p + b_p)$, where $\sigma(I'(T))$ is the standard deviation of the distribution of first passage times of a QIF neuron as it is depolarized past zero at rate $I'(T)$. In Figure S1D, we show that this standard deviation decreases with increasing $I'(T)$. We have $a_p + b_p > a_{min} + b_{min}$, so $\sigma(a_{min} + b_{min})$ is an upper bound on σ_p for all pools p. Thus, we have

$$\mathbb{E}[v_p] < \left(\frac{a_{max}}{a_{max}+b_{min}}\right)^2 \mathbb{E}\left[v_{p-1}\right] + \sigma(a_{min}+b_{min})^2. \qquad (19)$$

We consider the related equality, $\mathbb{E}[v_p] = \left(\frac{a_{max}}{a_{max}+b_{min}}\right)^2 \mathbb{E}\left[v_{p-1}\right] + \sigma(a_{min}+b_{min})^2$. This recurrence relation is of the form $x_p = Ax_{p-1} + B$, with $A = \left(\frac{a_{max}}{a_{max}+b_{min}}\right)^2$ and $B = \sigma(a_{min}+b_{min})^2$; as such, it describes the evolution of a linear discrete-time dynamical system. Solving the equality for $x_p = x_{p-1}$, we find that this system has a fixed point at $x_p = \frac{B}{1-A}$. Such a fixed point is asymptotically stable if $A \in (-1, 1)$, and we have $A = \left(\frac{a_{max}}{a_{max}+b_{min}}\right)^2 \in (-1, 1)$, so any solution to the equality approaches it asymptotically. Since $A > 0$, any solution to inequality (19) must stay strictly below the solution to the recurrence equation that is initialized from the same initial conditions. Therefore,

$$\lim_{p\to\infty} \mathbb{E}[v_p] < \frac{B}{1-A} = \frac{\sigma(a_{min}+b_{min})^2}{1-\left(\frac{a_{max}}{a_{max}+b_{min}}\right)^2},$$

where, as noted above, $\sigma(a_{min} + b_{min})$ decreases as b_{min} increases.
